# Genome-Wide Association Analysis of Heat Tolerance in F_2_ Progeny from the Hybridization between Two Congeneric Oyster Species

**DOI:** 10.3390/ijms25010125

**Published:** 2023-12-21

**Authors:** Mingyang Du, Zhuxiang Jiang, Chaogang Wang, Chenchen Wei, Qingyuan Li, Rihao Cong, Wei Wang, Guofan Zhang, Li Li

**Affiliations:** 1 CAS and Shandong Province Key Laboratory of Experimental Marine Biology, Center for Ocean Mega-Science, Institute of Oceanology, Chinese Academy of Sciences, Qingdao 266071, China; dumy@qdio.ac.cn (M.D.); jiangzx@qdio.ac.cn (Z.J.); wangcg@qdio.ac.cn (C.W.); weichenchen@qdio.ac.cn (C.W.); liqingyuan20@mails.ucas.ac.cn (Q.L.); rhcong@qdio.ac.cn (R.C.); wangwei@qdio.ac.cn (W.W.); gfzhang@qdio.ac.cn (G.Z.); 2 Laboratory for Marine Biology and Biotechnology, Laoshan Laboratory, Qingdao 266100, China; 3 University of Chinese Academy of Sciences, Beijing 101408, China; 4 National and Local Joint Engineering Laboratory of Ecological Mariculture, Institute of Oceanology, Chinese Academy of Sciences, Qingdao 266071, China; 5 Laboratory for Marine Fisheries Science and Food Production Processes, Laoshan Laboratory, Qingdao 266100, China; 6 Key Laboratory of Breeding Biotechnology and Sustainable Aquaculture, Institute of Hydrobiology, Wuhan 430072, China

**Keywords:** oysters, heat tolerance, GWAS, *Crassostrea gigas*, *Crassostrea angulata*, F_2_ progeny

## Abstract

As the world’s largest farmed marine animal, oysters have enormous economic and ecological value. However, mass summer mortality caused by high temperature poses a significant threat to the oyster industry. To investigate the molecular mechanisms underlying heat adaptation and improve the heat tolerance ability in the oyster, we conducted genome-wide association analysis (GWAS) analysis on the F_2_ generation derived from the hybridization of relatively heat-tolerant *Crassostrea angulata* ♀ and heat-sensitive *Crassostrea gigas* ♂, which are the dominant cultured species in southern and northern China, respectively. Acute heat stress experiment (semi-lethal temperature 42 °C) demonstrated that the F_2_ population showed differentiation in heat tolerance, leading to extremely differentiated individuals (approximately 20% of individuals die within the first four days with 10% survival after 14 days). Genome resequencing and GWAS of the two divergent groups had identified 18 significant SNPs associated with heat tolerance, with 26 candidate genes located near these SNPs. Eleven candidate genes that may associate with the thermal resistance were identified, which were classified into five categories: temperature sensor (*Trpm2*), transcriptional factor (*Gata3*), protein ubiquitination (*Ube2h*, *Usp50*, *Uchl3*), heat shock subfamily (*Dnajc17*, *Dnaja1*), and transporters (*Slc16a9*, *Slc16a14*, *Slc16a9*, *Slc16a2*). The expressional differentiation of the above genes between *C. gigas* and *C. angulata* under sublethal temperature (37 °C) further supports their crucial role in coping with high temperature. Our results will contribute to understanding the molecular mechanisms underlying heat tolerance, and provide genetic markers for heat-resistance breeding in the oyster industry.

## 1. Introduction

Oysters, as a significant aquaculture species globally, renowned for their nutritional value and salient role as a food resource, are widely farmed in Southeast Asia and North America [1,2,3]. As the world’s largest produced marine organism, oysters have become a traditional cornerstone industry in bivalve aquaculture in China, accounting for 80% of the total world production [4,5]. In addition to their economic value, oysters play an important role in the ecosystem [6,7]. Oysters can provide habitat for various fish and benthic invertebrates to maintain the biodiversity of ecosystems, and serve as a natural carbon sink by sequestering greenhouse gases from the atmosphere through shell production [8,9,10].

However, the frequent outbreaks of mass summer mortality worldwide have posed a serious threat to the development of the oyster industry, and have been documented in China [11], the United States [12], and Japan [13], resulting in significant economic losses. Previous studies have reported that the mass mortality of oysters is induced by multiple factors, such as high temperature [14], viral and bacterial infections [15], immune system dysregulation [16], and reproduction [17]. Among these, it is noteworthy that high temperature significantly induced the effects of other factors on all physiological processes of oysters, highlighting the urgent need to breed oyster strains with high-temperature tolerance [18,19,20]. Previous genetic improvement of resistance traits such as heat tolerance and disease resistance in oysters has mainly relied on traditional breeding methods, including selection [21], hybridization [22], and polyploidization [23], which are accompanied with long breeding cycles and low efficiency [24]. In addition, traditional breeding for resistance traits suffers from the difficulty of measuring individual phenotypes in vivo, resulting in slow progress. Genome-wide selection breeding and marker-assisted breeding are important approaches to enhancing breeding efficiency and overcoming bottleneck challenges in breeding for resistance traits [25], and have been limited to only a few species of shellfish and fish [26,27]. Therefore, investigating the molecular mechanisms underlying the heat tolerance and identifying the major genetic markers are essential for the molecular breeding of high heat-tolerant oysters.

*Crassostrea gigas* (*C. gigas*) and *Crassostrea angulate* (*C. angulate*) are two allopatric and dominant cultured oyster species, which naturally distribute in the North and South coasts of China bound by the Yangtze River and separately adapt to relative-cold (range from 5 °C to 25 °C) and -warm habitats (range from 13 °C to 28 °C) [28,29,30,31]. Previous research has confirmed that these two species exhibited differentiation in heat tolerance [32,33,34]. The semi-lethal temperature (LT50) was found to be significantly higher in *C. angulata* than in *C. gigas*, by approximately 1 °C, which indicated that *C. gigas* was more sensitive to high temperature [34]. The difference of one degree in the semi-lethal temperature leads to differential thermal tolerance physiological indicators, such as heart rate and standard metabolic rate, between these two species [34]. Differentiation of the resistant phenotypes and genetic divergence proved high potential to reveal the physiological and the genetic basis underlying heat tolerance [31,34]. Previous studies on the differential heat tolerance of *C. gigas* and *C. angulata* have mostly focused on comparing molecular and physiological traits between subspecies, including inter-species resequencing [31], identification of differentially expressed genes [35], and evaluation of heat-resistance physiological traits such as respiration rate and antioxidant enzyme activity [36]. However, we did not find any research on the genetic mapping of heat-resistance segregation populations derived from the hybridization of *C. gigas* and *C. angulata*.

The development of the whole-genome resequencing technology provides a large number of SNPs for genetic mapping, which enables whole-genome association and linkage analyses, such as genome-wide association analysis (GWAS), quantitative trait locus (QTL) mapping and bulked sample analysis (BSA) [37,38,39]. In aquaculture, GWAS, as a crucial genetic mapping method, has been widely applied to easily measurable quality traits such as growth [40,41,42] and nutrition [43,44], while studies on heat tolerance have been limited due to the difficulties in accurately assessing phenotypes. Previous studies of genetic mapping of heat tolerance in oysters have only been conducted on screening temperature-adapted genes between wild *C. gigas* and *C. angulata* [31] and the identification of the regulatory architecture of heat-responsive genes through eQTL in backcross populations between *C. gigas* and *C. angulata* [45], lacking the identification of genomic markers related to heat tolerance at the whole-genome level in hybrid populations between *C. gigas* and *C. angulata*. Heat tolerance is influenced by multiple genes and regulated by a complex network, which makes it challenging to accurately identify the related genes [46,47,48]. As the most accurate phenotype for evaluating the heat-resistance trait, time to death can result in the inability to validate critical genes from genetic mapping [49]. However, utilizing hybrid F_2_ populations with a similar genetic background but divergent tolerance derived from hybridization between species or populations with differentially resistant ability can significantly enhance the accuracy of genetic mapping, and validate the identified genes or loci through the parents [50]. Previous research on hybrid F_2_ populations derived from the hybridization between relatively heat-tolerant *C. angulata* and heat-sensitive *C. gigas* has mainly focused on the measurement of physiological phenotypes under heat stress [22], and has not been utilized as an excellent research material to reveal the molecular mechanisms of heat response and identify genetic markers underlying heat tolerance.

In this study, we conducted whole-genome resequencing and GWAS to identify candidate loci and genes which are associated to the heat tolerance of the F_2_ populations of hybrids (AG × AG). Based on populations with differential tolerance, we identified 18 significant SNPs and 26 candidate genes, among which 11 genes are related to a series of thermally adapted biological processes. The results offer a deeper understanding of the genetic basis of heat tolerance, facilitate investigations into the molecular regulation underlying heat response, and provide valuable genetic markers for the improvement of resistance in oyster industry.

## 2. Results

### 2.1. Heat Tolerance Differentiation

**Figure 1:**
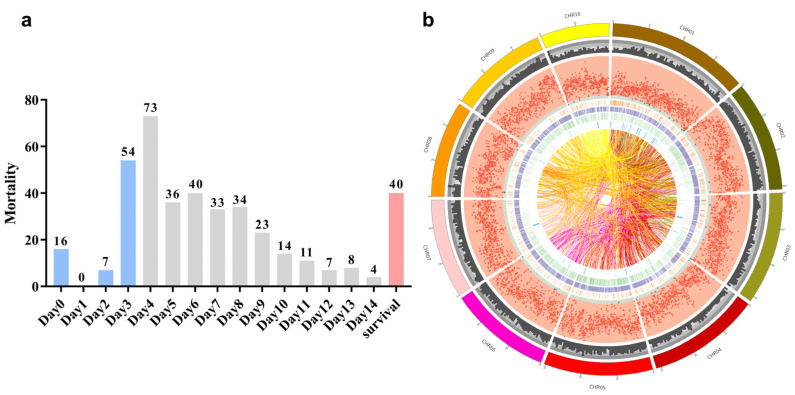
The plot of the daily number of deaths during heat stress and the Circos plot of an overview of the overall analysis results. (**a**) The plot of the daily number of deaths during heat stress. Each bar represents the number of deaths per day. The blue bar represents the “heat-sensitive (HS)” group, while red bar represents the “heat-tolerant (HT)” group. (**b**) The Circos plot of an overview of the overall analysis results. The genome information at different levels was represented using several circles, with each circle representing a specific aspect. Starting from the outermost circle, the length of each chromosome is depicted. The frequency histogram represents the gene density in different regions of the chromosome, and the scatter plot depicts the SNP density in different regions of the chromosome. The innermost circles depict different types of structural variations, with the orange line representing DUP (tandem duplication), the purple line representing DEL (large deletion), the green line representing INS (insertion), and the blue line representing INV (inversion). The connecting lines in the inner circle represent the BND (chromosome translocation) structural variation and its location on the chromosome.

Heat tolerance was evaluated based on the time to death, individuals with poor heat tolerance were more susceptible to mortality during exposure to extreme heat. The number of deaths per day is shown in Figure 1a. On the first day of the heat stress experiment, 4% (16) of individuals died, and over the next two days (Day 1, Day 2), only seven individuals died. On Day 3 and Day 4, nearly 30% (127) of the individuals died. In the following four days (Day 5–Day 8), the mortality rate was relatively stable, with approximately 35% (143) of the individuals dying. From Day 9 to Day 14, the number of deaths gradually decreased each day, with 40 individuals still surviving on Day 14. The forty oysters which died earlier (16 at Day 0; 0 at Day 1; 7 at Day 2; 17 at Day 3; total 40) were considered as the “heat-sensitive (HS)” group, the last surviving 40 oysters were considered as the “heat-tolerant (HT)” group.

### 2.2. Sequencing and Genotyping

We have preliminarily obtained 5,291,912 SNPs and 645,661 indels. Then, after filtering with the PLINK software (v2.0), a total of 2,744,222 single nucleotide polymorphisms (SNPs) and 362,292 insertions/deletions (indels) were obtained for subsequent GWAS analysis (Table S1). The positional information of the SNPs in each sample is shown in Figure S1. The gene density and SNP density in different regions of the chromosome within a specific window and other information are shown in Figure 1b. The sequencing data in this study have been deposited into the Sequence Read Archive (SRA) BioProject, under the accession number PRJNA984967.

### 2.3. Linkage Disequilibrium (LD) and Population Structure 

**Figure 2:**
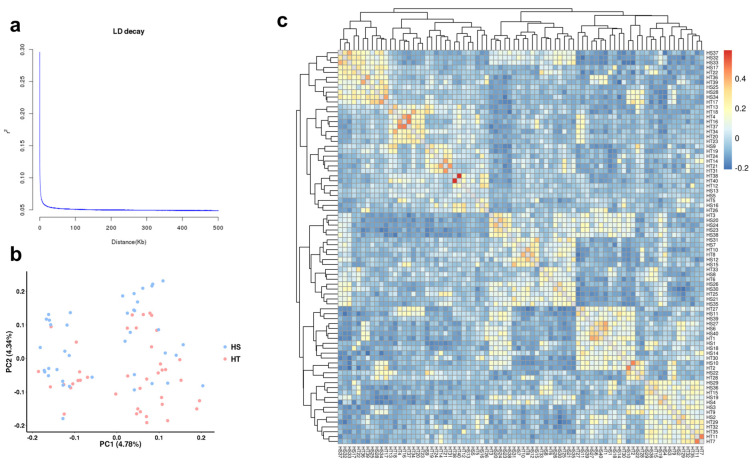
Population structure and linkage disequilibrium (LD) decay analysis. (**a**) LD decay. The *x*-axis represents the physical distance between SNPs, while the *y*-axis shows the average r^2^ values for each distance bin. (**b**) Principal components analysis (PCA). The blue and red dots represent individuals from the heat-sensitive (HS) and heat-tolerant (HT) groups, respectively. (**c**) Kinship among the resequencing samples. The color-coded matrix shows the pairwise kinship coefficients, with warmer colors indicating higher levels of relatedness.

When the distance between loci was approximately 500 bp, the r^2^ value decreased to 0.1, indicating a relatively low degree of LD between the loci (Figure 2a). Principal component analysis (PCA) revealed a subtle population stratification within the population, which could lead to false positive associations between genetic variants and disease in GWAS analysis (Figure 2b). To address this potential confounding factor in GWAS, a kinship matrix was included as a random effects covariate matrix (K matrix) in the analysis (Figure 2c). The frequency distribution plot of kinship could provide a clear visualization of the main distribution range of the pairwise relatedness values among the studied samples, which could aid in the identification of samples with extreme kinship values. Based on the cross-validation error rate line plot in the Admixture analysis (Figure S2), the CV error reaches its minimum when K = 2. The genetic composition matrix of the samples corresponding to this K value, with the last cluster value removed, could be used as the fixed effect covariate matrix (Q matrix) in the GWAS analysis to control for false positives caused by population structure.

### 2.4. Genome-Wide Association Study (GWAS)

**Figure 3:**
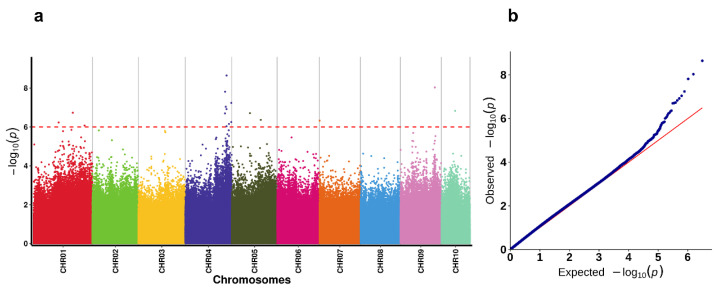
The Manhattan and quantile–quantile (QQ) plots from GWAS analysis. (**a**) Manhattan plots. The red dashed line represents the genome-wide significant threshold (−log_10_(1 × 10^−6^) = 6). (**b**) Quantile–quantile (QQ) plots. The red line represents the expected distribution of −log_10_(1 × 10^−6^) values under the null hypothesis, while the deviations from this line reflect the *p*-value from which the regression of the phenotype on the SNP deviates from what is expected by chance.

After considering the kinship and population structure of the heat-tolerant (40 individuals) and heat-sensitive (40 individuals) groups, we obtained a Manhattan plot and quantile–quantile plot of heat tolerance (Figure 3). According to the set threshold (−log_10_*p* > 6), we identified a total of 18 significant SNPs across 10 chromosomes (Table 1). A significant peak was observed on chromosome 04, with the most significant locus being CHR04_55554566 (*p* = 2.24969 × 10^−9^).

We screened the genes within 50 kb of the 18 significantly identified SNPs, and 26 genes were speculated to be associated with heat tolerance (Table 2), including Ubiquitin Conjugating Enzyme E2 H (*Ube2h*), Ubiquitin C-Terminal Hydrolase L3 (*Uchl3*), Ubiquitin Specific Peptidase 50 (*Usp50*), Transient Receptor Potential Cation Channel Subfamily M Member 2 (*Trpm2*), DnaJ Heat Shock Protein Family Member A1 (*Dnaja1*), DnaJ Heat Shock Protein Family Member C17 (*Dnajc17*), Solute Carrier Family 16 Member (*Slc16a2*, *Slc16a9*, *Slc16a12*, *Slc16a14*), GATA Binding Protein 3 (*Gata3*), etc. These candidate genes are included in various pathways with different biological functions, such as metabolic pathways, ubiquitination, temperature sensing pathways, heat shock pathways, and transport protein pathways.

### 2.5. qRT-PCR Validation Results

**Figure 4:**
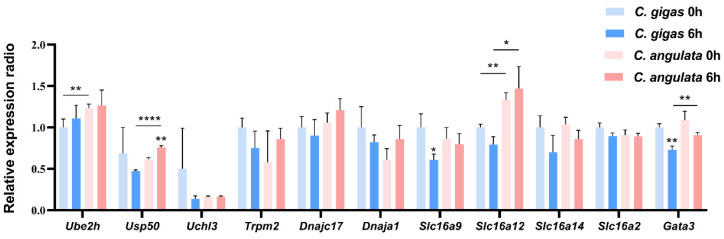
The relative expression radio of candidate genes in heat stress experiment (37 °C) of *C. gigas* and *C. angulata*. The light blue and blue bars represent the gene expression levels of *C. gigas* under basal (0 °C) and heat stress (37 °C) conditions, respectively. The light red and red bars represent gene expression levels of *C. angulata*. The error bars represent the SD. Significant differences among groups were marked with * *p* < 0.05, ** *p* < 0.01, **** *p* < 0.0001.

The qRT-PCR (Figure 4) indicated that the ubiquitination process-related genes *Ube2h* showed an upward trend in both *C. gigas* and *C. angulate* after heat stress (37 °C 6 h), and the baseline (0 h) expression of these genes in *C. angulate* was significantly higher than that in *C. gigas*. In contrast, *Uchl3*, *Usp50* showed a decrease after heat stress in *C. gigas*. The expression of *Usp50* significantly increased after heat stress in *C. angulata*, and the expression level was significantly higher than that in *C. gigas.* The expression of *Trpm2*, *Dnajc17*, *Dnaja1*, and *Slc16a12* exhibited an opposite response pattern between the two species, with a decreasing trend observed in *C. gigas* after heat stress, while an increasing trend was observed in *C. angulata*. The baseline expression and expression after heat stress of *Slc16a12* in *C. angulata* was significantly higher than that in *C. gigas*. The expression of *Slc16a9*, *Slc16a14*, *Slc16a2*, and *Gata3* showed a decreasing trend after heat stress, but the decrease was more pronounced in *C. gigas*. After heat stress, the expression of *Gata3* was significantly higher in *C. angulata* compared to *C. gigas*.

## 3. Discussion

In this study, the F_2_ progeny from the hybridization between *C. angulata* and *C. gigas* were subjected to an acute heat stress experiment (42 °C). The results showed nearly 20% (77) individuals died within four days under heat stress, and 10% (40) individuals were still alive after 14 days, indicating a divergence in the heat tolerance between the individuals that died earlier and those that survived until the end of the experiment. The phenomenon of trait segregation in the F_2_ offspring of a hybrid may be due to the occurrence of the Mendelian inheritance principle during F_1_ self-crossing, which has been widely applied for fine mapping of quantitative trait loci in plants and fish [50,51]. By conducting quantitative trait locus (QTL) mapping in the F_2_ population of zebrafish (*Metriaclima zebra*), researchers have identified loci that are significantly associated with pigment deposition traits [51]. It has been reported in cucumber (*Cucumis sativus* L.) that a high-carotenoid content variety has been selected through F_2_ population trait segregation [52]. In watermelons (*Citrullus lanatus*), the major QTL associated with the carpel number trait was identified using bulked segregant analysis (BSA-seq) in the F_2_ population [53] The phenomenon of trait segregation (differential heat tolerance) in F_2_ populations provided a prerequisite for accurately identifying heat tolerance-related loci using GWAS.

GWAS results may be biased by population stratification and familial relatedness, potentially leading to false positive associations [54,55]. Based on quantile–quantile (QQ) plots of four models, GLM models were deviated significantly from expected distribution values, thereby we determined the MLM (QK) model with two covariates which had lower false positive rates and more accurate results. LD analysis was a coefficient used to measure the non-random association of alleles at two or more loci on a chromosome. The squared correlation coefficient (r^2^) was commonly used to quantify the strength of LD, and it rapidly decreased as the distance between SNPs increased, which is consistent with several studies on aquatic animals, such as heat stress in interspecific backcross progenies of channel catfish, metal accumulation in oysters, and the association analysis of fatty acid content in Atlantic salmon [44,56]. Additionally, In *C. gigas* and *C. angulata*, when the r^2^ value decreased to 0.1, the distance between loci decreased to approximately 600 bp and 1000 bp, respectively [57]. In our study, the LD decay distance of F_2_ population was approximately 500 bp. These findings indicated that hybridization of the F_2_ population does indeed break down linkage disequilibrium (LD) to a certain extent, leading to increased independence of genetic variation between different loci.

A total of 18 SNPs and the associated 26 genes were identified in this study, and 11 genes were considered to be potentially associated with the thermal resistance and categorized into five functional groups, including temperature sensors, transcription factors, heat shock proteins, ubiquitination processes, and transmembrane transporters. Temperature sensors, as signal generators, are essential for detecting environmental changes by sensing variations in ambient temperature and subsequently regulating downstream gene expression, as shown in Drosophila brain thermoreceptor cells [58]. The relevant gene *Trpm2* was identified on chromosome 01, which belongs to the transient receptor potential melastatin (TRPM) ion channel subfamily. TRPM is non-selective Ca^2+^ permeable cation channel that regulates ion homeostasis and is a critical cellular sensor for various biological signaling pathways, such as cell apoptosis and multiple immune functions [59]. It is noteworthy that TRPM channels are regulated by ambient temperature, with warm temperatures (>35 °C) directly inducing TRPM2 channel gating [60]. In our previous studies, *Trpm2* has also been identified as environment responsive and a selected gene based on resequencing and comparison of the transcriptomes of *C. gigas* and *C. angulata* [31]. The expression pattern of *Trpm2* in *C. gigas* and *C. angulata* showed that *C. angulata*, with relative high heat tolerance, exhibits an increase in expression levels under heat stress conditions, which further supported the involvement of the *Trpm2* in mediating heat tolerance differentiation. Therefore, the identification of *Trpm2* in the AG × AG F_2_ population in this research confirms its critical role in heat stress resistance ability, which may inhibit cell apoptosis via the opening of Ca^2+^ channels.

GATA3, a dual zinc finger transcription factor, was identified in chr 04 in this study, which plays an important role in regulating cellular immunity by promoting the T cell differentiation [61]. Mass summer mortality is often accompanied by a weakening of the immune response system due to high temperatures, which enhances the infections by parasites such as *Vibrio* [62]. The research on the immune response of *C. gigas* after infection with *V. splendidus* found that the expression of GATA3 and other immune-related proteins is significantly upregulated, indicating the key role of GATA3 in defending the invading pathogens [63,64]. The expression results of *Gata3* were influenced by high temperature and *C. angulata* was significantly higher than *C. gigas* after heat stress, which supports that *Gata3* has temperature responsiveness and is involved in the immune activity of oysters under thermal stress. Therefore, GATA3 plays an important role in the immune invasion of oysters enhanced by high temperature.

In addition, genes related to protein stability pathways were identified, including the ubiquitination process (*Ube2h*, *Uchl3*, *Usp50*) and heat shock genes (*Dnajc17* and *Dnaja1*). When the environmental temperature exceeds the regulatory capacity of the organism, a range of adaptive physiological and cellular mechanisms will be triggered, along with the accumulation of undergo misfolding or remain unfolded proteins inside the cells, leading to endoplasmic reticulum (ER) stress, subsequently causing cellular autophagy and apoptosis [65]. The balance between protein synthesis, folding, and degradation is crucial for maintaining a functional proteome. There are two key pathways to maintain the balance from stress conditions, the ubiquitin-proteasome system (UPS) and the heat shock response (HSR) typically represent the cellular degradation and protein homeostasis machinery [66,67]. Three genes (*Ube2h*, *Uchl3*, *Usp50*) related to the ubiquitination process were identified in this study. UBE2H is a ubiquitin-conjugating enzyme E2 that facilitates the covalent attachment of ubiquitin moieties to protein substrates, thereby marking them for degradation via the ubiquitin–proteasome system [68]. There have been reports in turbot (*Scophthalmus maximus*) confirming that UBE2H responds to high-temperature stress and has an antagonistic relationship with p53, which plays a crucial regulatory role in controlling cell cycle arrest and apoptosis in response to cellular stress [69]. Ubiquitin C-terminal hydrolase L3 (UCHL3) is an important member of the ubiquitin C-terminal hydrolase family, exercising DNA damage repairing and homeostasis maintaining functions under heat stress [70]. Additionally, it has been reported that the downregulation of the ubiquitination-related gene *Usp50* can suppress the expression of mitochondrial Carnitine Palmitoyl transferase 1A (CPT1A), leading to the decrease of fatty acid beta-oxidation and energy supply, which play an important role in energy supply during heat stress [71]. Based on the expression results, genes related to the ubiquitination process were highly expressed in relatively heat-tolerant *C. angulata* after heat stress. This also underscores the connection between ubiquitination-related genes and heat tolerance, which may enhance the regulation of damaged proteins, increase DNA repair ability and energy production, and ultimately contribute to stronger heat tolerance.

Fortunately, we identified two genes directly related to heat shock response (*Dnajc17* and *Dnaja1*) on chromosome 02, which is another kind of balance, maintaining pathways activated by different stressors and generally accompanied by the increased expression of heat shock proteins [72,73]. The *Dnaj* family is a subfamily of *Hsp40* that typically functions as accessory proteins to HSP70, assisting in the folding, stabilization, and unfolding of client proteins [74]. In the yellow croaker and catfish, the heat-tolerant and heat-sensitive groups have identified the DNAJ family genes using GWAS, suggesting the strong association of DNAJ with heat tolerance [56,75]. Under heat shock conditions, DNAJ can interact with damaged proteins to protect their substrates against conformational damage, and promote the repair or degradation function to prevent aggregation and prevent formation of toxic inclusion bodies, which further maintain the intracellular homeostasis from disturbance [76]. A short-term and long-term heat stress experiment on honeybees (*Apis cerana*) has suggested that the stress-induced response of DNAJA1, and the decrease of heat tolerance after the knockdown of DNAJA1, proved its key role in heat stress resistances [77]. There are few reports on DNAJC17, but as a member of the heat shock protein (HSP40) family, it is speculated that may regulate cell proliferation [78]. Similar to *Trpm2*, the differential expression pattern of Dnajc17, Dnaja1 in *C. gigas*, and *C. angulata* after heat stress suggested that the upregulation of these genes in *C. angulata* may play a critical role in mitigating the effects of heat stress. This is consistent with previous findings that, in response to heat stress, *C. angulata* has lower basal expression levels but stronger plasticity in the expression of HSPs [79]. Therefore, these genes may act as molecular chaperones to assist heat-damaged proteins in performing normal functions.

Energy metabolism plays a crucial role in the stress adaptation and tolerance response of organisms, as extra energy is required to recover from and maintain the internal balance [80]. As the transporter proteins, the SLC family plays a significant role in maintaining the cellular metabolism and energy supply [81,82,83]. The identified genes (*Slc16a2*, *Slc16a9*, *Slc16a12*, *Slc16a14*) in our research belong to the *Slc16a* family, which is a group of transmembrane transport proteins mainly involved in the transport of organic acids such as lactate and pyruvate [81,84]. Pyruvate is a crucial metabolite in glucose metabolism, which is key source of biological energy. In previous studies, we found differential expression genes in the *Slc* superfamily between China’s north and south coastlines cultured *Crassostrea ariakensis*, suggesting that the *Slc* family, such as *Slc16a2*, plays a critical role in the temperature adaptation [31]. And it has also been identified in the RNA-Seq of high-temperature-tolerant differentiated corals [85]. Additionally, comparative analysis of *C. gigas* and *C. angulata* revealed that *C. angulata* exhibits stronger energy metabolism capability under heat stress [86]. In this study, the *Slc* family genes were found to be expressed at higher levels in *C. angulata* than *C. gigas* after heat stress, which may enhance their capacity for carbohydrate metabolism. This provides evidence for the important role of the *Slc* family in heat tolerance.

## 4. Materials and Methods

### 4.1. AG × AG F_2_ Populations Construction

The experimental design of spawning is referred to our previous study [32]. The parents of F_1_ populations were wild *C. gigas* and *C. angulata*, which were collected from their respective natural habitats Qingdao (119°30′ E, 35°44′ N) and Xiamen (118°4′ E, 24°33′ N) in China [32]. We mixed the eggs from 30 mature female *C. angulata* and divided them into 30 beakers. The sperm from 30 mature male *C. gigas* was separately used to fertilize the eggs in each beaker. The fertilized eggs were cultured in an indoor hatchery (24 °C) in Laizhou City (119°30′ E, 37°18′ N, Shandong Province, China) and larvae were cultured in the outdoor pond (18–20 °C) from February 2021 to June 2021. Then, the four months old F_1_ progeny were transported to the sea of Muping city (121°60′ E, 37°39′ N, Shandong Province, China) for cultivation. In the second year (February 2022), after the F_1_ population reached sexual maturity, 30 males and 30 females were mated using the same method as the F_1_ in Laizhou City. The resulting F_2_ progeny were also transported to the sea of Muping city and cultured for nine months, then brought to Qingdao for subsequent experiments in November 2022.

### 4.2. Heat Stress Experiment and Sample Collection

Four hundred nine months old oysters from AG × AG F_2_ populations were cleaned and reared in a 500 L tank with sand-filtered and aerated seawater (water temperature 15 ± 2 °C) for one week to minimize the potential impact of environmental factors on subsequent experiments. After the acclimation period, 400 oysters were placed into semi-lethal temperature (42 °C) seawater for 1 h [34]. The water temperature was maintained by a variable frequency heating rod. After the acute heat stress experiment, oysters were put back into the 500 L tank (water temperature15 ± 2 °C) with continuous aeration. The dead oysters were removed in time and we recorded the number of deaths daily for fourteen days. The gills of the oysters were sampled and placed in liquid nitrogen, and stored in a −80 °C freezer. The 10% oysters (40 individuals) which died first and 10% survivals (40 individuals) (at Day 14) were considered as “heat-sensitive (HS)” and “heat-tolerant (HT)” groups. Fifteen wild *C. gigas* and *C. angulata* were exposed to sublethal temperature (37 °C) seawater for six hours. After the heat exposure, gill tissues were collected from each individual and stored in a −80 °C refrigerator. Moreover, the same number of samples were collected from *C. gigas* and *C. angulata* without the heat stress treatment (0 h) as control. The detail of experiment design is shown in Figure S3.

### 4.3. Sequencing, Genotyping, and Quality Control

The genomic DNA of 40 oysters in the HT group and 40 oysters in the HS group were isolated from gills using the TIANamp Marine Animals DNA Kit (TIANGEN, Beijing, China). The quality of the extracted DNA was evaluated using 1% agarose gel electrophoresis, and the concentration was determined using a Nanodrop 2000 spectrophotometer (ThermoFisher Scientific, Walham, MA, USA). The flow of genome resequencing was as follows: the genomic DNA was randomly sheared into short fragments using enzymes, followed by end repair. Subsequently, dA-tails and sequencing adapters were ligated to both ends of the DNA fragments. After purification with AMPure XP magnetic beads (ThermoFisher), the DNA fragments with adapters were selectively amplified by PCR within the 300–400 bp range. The DNA libraries were sequenced using the Illumina NovaSeq 6000 platform by Genedenovo Biotechnology Co., Ltd. (Guangzhou, China). The data were aligned to the Pacific oyster genome (GenBank accession no. GCA_011032805.1) using the alignment software BWA-MEM (Version 0.7.12) [87,88]. Further quality control was performed by using the PLINK software (v2.0) with the following criteria. (1) Non-biallelic sites. (2) Sites with a minor allele frequency (MAF) less than 0.05. (3) Sites with a missing rate greater than 0.3. (4) Sites with a heterozygosity rate greater than 0.8.

### 4.4. Linkage Disequilibrium and Population Structure Analysis

In addition to the quality control criteria mentioned earlier, marker independence screening is also performed based on the linkage disequilibrium (LD) between markers. In this analysis, we used the PLINK software (v2.0) [89] to remove one of the markers in pairs with an r^2^ greater than 0.2 within a 10 nt window and 100 kb step size (the marker with the later physical position was removed). The GCTA software (v1.93.2) [90] was used to perform principal component analysis (PCA) and to obtain the kinship matrix of pairwise relationships between samples based on the selected SNP markers. The variance explained by each principal component (PC) and the sample scores in each PC were obtained. We employed the PopldDecay software (v3.41) [91] to compute the linkage disequilibrium (LD) (r^2^) between all possible pairs of markers, and visualized the resulting LD decay curve as a function of genetic distance. The degree of LD between two genetic variants was indicated by r^2^, where a value of 0 indicates no LD, while a value of 1 represents complete LD. The population structure analysis used the Admixture software (v1.3) [92]. We assumed the number of subgroups (K value) to be 1–9 and performed clustering analysis. The optimal number of subgroups was determined based on the cross-validation error (CV error) rate, with the K value corresponding to the minimum CV error rate being the optimal number of subgroups.

### 4.5. Genome-Wide Association Analysis and Candidate Genes Annotation

Based on the results of PCA analysis, we used the GEMMA software (v0.98.1) [93] to perform GWAS analysis using the commonly used GLM and MLM model. The kinship matrix of pairwise relationships and population structure between samples, calculated using GCTA and the Admixture software, was included in the GWAS MLM model. The formulas used in this study are as follows:

y = Xα + Qβ + Kµ + e(1)

where y is the phenotype vector (heat tolerance), α is the genotype effect vector, β is the fixed effect vector including the effect of the SNPs, and µ and residual vector e are the random effect vectors. The matrices X, Q, and K are incidence matrices relating the effect vectors with the individual phenotypes in y. For each SNP locus, we test whether α is equal to zero, and the probability value *p* of α being zero is used to measure the degree of association between the marker genotype and the phenotype. The smaller the *p*-value, the smaller the probability of α being zero, indicating a stronger association between the marker and the trait. The density plot and Manhattan plot of the −log_10_(*p*-value), as well as a quantile–quantile (QQ) plot of the original *p* values for each SNP, were generated using the R package CMplot v3.6.0.

Threshold as −log_10_*p* > 6 was set to identify significant SNPs and we scanned the genome region located approximately 50 kb upstream and downstream of significant SNPs for candidate genes. The nearest coding sequences to each SNP were identified, retrieved, and annotated using BLAST analysis against the protein database NR/Swiss-Prot dataset [87].

### 4.6. qRT-PCR Experiment

The total RNA of 60 oyster gill tissues from the sublethal temperature (37 °C) heat stress experiment was extracted using Trizol reagent (Vazyme Biotech, Nanjing, China). First-strand cDNA was synthesized using HiScript III RT SuperMix for qPCR (Vazyme Biotech) and subsequent qPCR experiments were performed using Taq Pro Universal SYBR qPCR Master Mix (Vazyme Biotech) on the ABI 7500 FAST system (Applied Biosystems, Waltham, MA, USA). Each experimental group (control and heat stress groups) comprised three independent biological replicates, which were prepared by pooling the cDNA samples of five oysters in equal amounts. The resulting mixtures were subjected to three technical replicates to ensure the accuracy and reproducibility of the measurements. The primers were designed using the Primer5 software (Table S2). All samples were examined in technical triplicates, and the Ef-1α gene was used as an internal control [79]. A detailed comparison between two species has been previously described [79]. All statistical analyses were performed using GraphPad Prism version 8.0.2. The statistical analyses conducted in this study employed two-way ANOVA analysis and subsequent Bonferroni multiple comparison test to determine the significant differences between different treatments. Significant differences between groups were marked with “*” for *p* < 0.05, “**” for *p* < 0.01, and “****” for *p* < 0.0001

## 5. Conclusions

In summary, we identified 18 significant SNPs and 26 candidate genes associated with heat tolerance by GWAS in F_2_ progeny with divergent heat tolerance derived from the hybridization between *C. angulata* (AA) and *C. gigas* (GG). The associated genes included temperature sensing (*Trpm2*), transcription factor (*Gata3*), protein stabilization (*Ube2h*, *Usp50*, *Uchl3* and *Dnaj*), and Solute Carrier Family (*Slc16a2*, *Slc16a9*, *Slc16a12*, *Slc16a14*) genes. The qRT-PCR results of the relatively heat-sensitive *C. gigas* and the relatively heat-tolerant *C. angulata* further support the important role of these key genes in heat tolerance, which may be involved in multiple mechanisms such as Ca^2+^ influx, transcriptional signal regulation, protein degradation or folding, and transport of metabolites pyruvate to regulate the thermal response of marine organisms. Our findings provided new insights into the regulatory network underlying the thermal divergence of marine organisms, and produced genetic markers for heat-resistance breeding in oysters and aquaculture. However, the effectiveness of molecular markers still requires validation through multi-generational breeding.

## Figures and Tables

**Table 1:** The significantly identified SNPs between differential heat-tolerant F_2_ populations from the hybridization between *Crassostrea angulata* × *C. gigas*.

	Gene ID	Start	End	Gene Name	Gene Annotation	Classification
CHR01	g01534	33,529,362	33,551,395	*Megf10*	Multiple epidermal growth factor-like domains protein	-
	g01537	33,606,084	33,608,607	*Cav2*	Caveolin-2	-
	g03038	68,399,030	68,404,722	*Trpm2*	Transient receptor potential cation channel M member 2	temperature sensors
CHR02	g06281	45,304,077	45,345,630	*Comp*	Cartilage oligomeric matrix protein	-
	g06282	45,365,916	45,367,651	*Dnajc17*	DnaJ homolog subfamily C member 17	heat shock proteins
	g06283	45,373,304	45,375,343	*Dnaja1*	DnaJ homolog subfamily A member 1	heat shock proteins
	g06284	45,376,540	45,393,197	*Mettl3*	N6-adenosine-methyltransferase catalytic subunit	-
	g06286	45,393,469	45,403,190	*Cpsf6*	Cleavage and polyadenylation specificity factor subunit 6	-
CHR04	g12537	53,406,861	53,433,358	*C1ql4*	Complement C1q-like protein 4	-
	g12538	53,437,493	53,454,981	*Ube2h*	Ubiquitin-conjugating enzyme E2 H	ubiquitination processes
	g12549	53,638,138	53,654,138	*Cttnbp2*	Cortactin-binding protein 2	-
	g12592	54,489,394	54,502,007	*Usp50*	Ubiquitin carboxyl-terminal hydrolase50	ubiquitination processes
	g12597	54,554,609	54,560,671	*Uchl3*	Ubiquitin carboxyl-terminal hydrolase isozyme L3	ubiquitination processes
	g12599	54,566,743	54,573,426	*Prpf18*	Pre-mRNA-splicing factor 18	-
	g12652	55,507,413	55,508,138	*C1qtnf4*	Complement C1q tumor necrosis factor-related protein 4	-
	g12653	55,509,496	55,514,690	*Grhpr*	Glyoxylate reductase/hydroxypyruvate reductase	-
	g12658	55,571,045	55,583,506	*Nek4*	Serine/threonine-protein kinase Nek4	-
	g12825	58,764,950	58,784,477	*Gata3*	Transcription factor GATA-3	transcription factors
CHR05	g15391	38,958,891	38,967,233	*Slc16a9*	Monocarboxylate transporter 9	transmembrane transporters
	g15392	38,973,035	38,985,570	*Slc16a14*	Monocarboxylate transporter 14	transmembrane transporters
	g15393	38,988,858	38,999,293	*Slc16a12*	Monocarboxylate transporter 12	transmembrane transporters
	g15394	39,005,142	39,014,052	*Slc16a2*	Monocarboxylate transporter 2	transmembrane transporters
CHR07	g20019	305,324	309,093	*Yabd*	Deoxyribonuclease YabD	-
	g20021	330,650	333,412	*Tatdn2*	Deoxyribonuclease TATDN2	-
CHR09	g28376	46,302,989	46,305,847	*Scop1*	Serine rich endogenous peptide 1	-
	g28379	46,334,670	46,354,389	*Itih4*	Inter alpha-trypsin inhibitor, heavy chain 4	-

A1: minor allele; A0: major allele; FDR: false discovery rate; beta: beta estimates which are the effect sizes of SNPs.

**Table 2:** The information of the candidate genes within 50 Kb of the significant SNPs.

SNP	Chr	Pos	A1	A0	FDR	Beta	*p* Value
CHR01_33558322	CHR01	33,558,322	A	T	0.130237	−0.34032	5.87 × 10^−7^
CHR01_52587047	CHR01	52,587,047	C	T	0.062233	0.524834	1.85 × 10^−7^
CHR01_68449911	CHR01	68,449,911	T	G	0.163389	0.405082	8.42 × 10^−7^
CHR02_45368198	CHR01	45,368,198	C	A	0.169521	−0.50435	9.82 × 10^−7^
CHR04_53398632	CHR04	53,398,632	C	G	0.062233	−0.52111	2 × 10^−7^
CHR04_53638776	CHR04	53,638,776	T	G	0.015796	−0.41998	1.53 × 10^−8^
CHR04_54528444	CHR04	54,528,444	C	A	0.056362	−0.33407	9.08 × 10^−8^
CHR04_55502224	CHR04	55,502,224	C	T	0.168788	−0.6035	9.24 × 10^−7^
CHR04_55503359	CHR04	55,503,359	A	T	0.062003	−0.62427	1.2 × 10^−7^
CHR04_55554566	CHR04	55,554,566	G	A	0.006984	0.408852	2.25 × 10^−9^
CHR04_58789597	CHR04	58,789,597	A	T	0.145299	−0.38664	7.02 × 10^−7^
CHR04_62006586	CHR04	62,006,586	A	T	0.044535	0.35255	5.74 × 10^−8^
CHR04_62006599	CHR04	62,006,599	A	T	0.130237	0.329153	5.56 × 10^−7^
CHR05_24461145	CHR05	24,461,145	T	C	0.062233	0.422095	1.95 × 10^−7^
CHR05_38969828	CHR05	38,969,828	G	C	0.122076	−0.46043	4.33 × 10^−7^
CHR07_343905	CHR07	343,905	C	A	0.122917	0.59167	4.75 × 10^−7^
CHR09_46322969	CHR09	46,322,969	T	C	0.014379	0.443599	9.26 × 10^−9^
CHR10_18611994	CHR10	18,611,994	T	C	0.062233	−0.61093	1.48 × 10^−7^

## Data Availability

The genome resequencing data was deposited in the GenBank with accession number PRJNA984967.

## Supplementary Materials

The following supporting information can be downloaded at: https://www.mdpi.com/article/10.3390/ijms25010125/s1.
