# Supplementary material for: Genome-Wide Association Analysis of Heat Tolerance in F2 Progeny from the Hybridization between Two Congeneric Oyster Species
Source: Int J Mol Sci. 2023 Dec 21;25(1):125. doi: 10.3390/ijms25010125 (PMC10778899; doi:10.3390/ijms25010125)
Supplement: Supplementary file 1 [file ijms-25-00125-s001.zip › ijms-2762246-supplementary.pdf]

Table S1. Statistical analysis of marker filtering results.

Variation	All	Keep
Total	3106514	3104571
SNP	2744222	2742481
Indel	362292	362090

Variation: types of mutations; All: all sites before filtering; Keep: remaining sites after filtering.

Table S2. Primer sequences used in qRT-PCR Experiment.

Primer name	Sequences (5'-3')
Ube2h-F	TCGCATGGTTACACTTGG
Ube2h-R	TGTTTTCCGTTCGCAGA
Usp50-F	ATGTAGGCTGTGAAAATCCTTC
Usp50-R	TGTTCTGTCAACTGTGTCTGGT
Uchl3-F	ATCCAGCATTCCATCACG
Uchl3-R	AGAGGAGGAGGAGCATCAG
Trpm2-F	TCAGATTCCTGTTTTTGGC
Trpm2-R	CTCATTGTCGGTCTCTCCTT
Dnaja1-F	GTCCGAGTTTTACTTGTCTGA
Dnaja1-R	AGCGTGCCTTCCTTTCT
Dnajc17-F	GAAATGAGAAAAACAGGCAG
Dnajc17-R	AAACGCAAAGAATGGACC
Slc16a9-F	AAATGGGGTAGTATGTGCC
Slc16a9-R	AGAAGCGTTGAAATGTCTTG
Slc16a12-F	CAGAGACAAATAGCACGCA
Slc16a12-R	AGCCAACAAAAGGACCG
Slc16a14-F	CTTCTCGTAACCTTCGCC

Slc16a14-R	AAACATCAGACCCCCCA
Slc16a2-F	TTCTGAGGAAACCACGGA
Slc16a2-R	GGGACCAGCACACAAATAG
Gata3-F	TGTGGAGGAGGGATGGG
Gata3-R	TGTAAGCGGGCGGTT

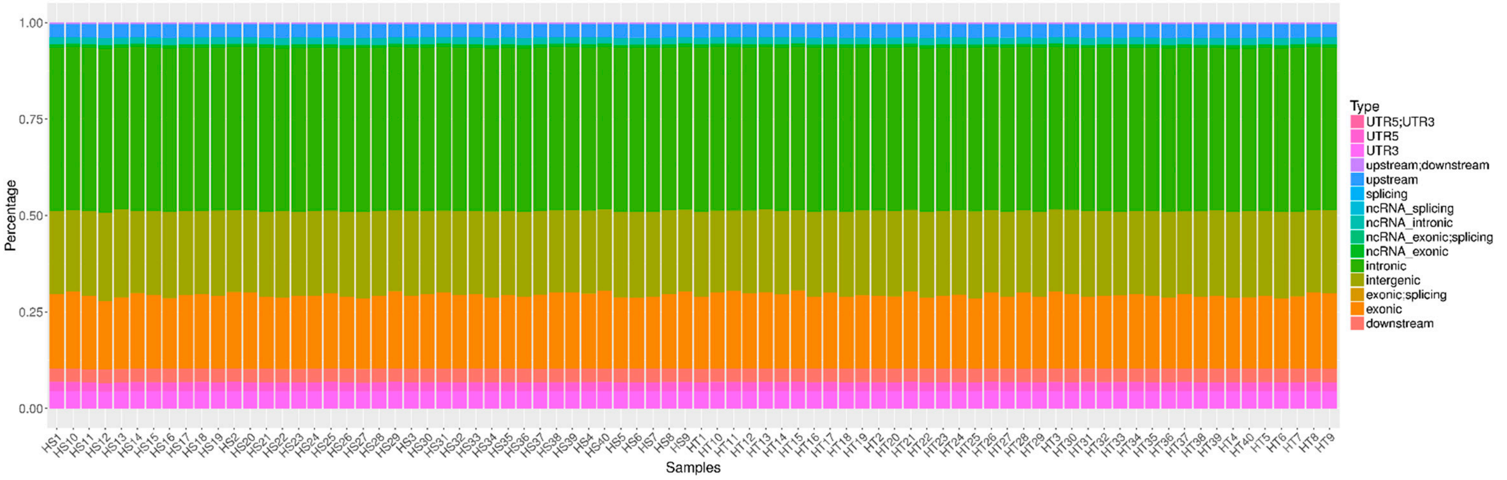

Figure S1. The graphical representation of the genomic position information of the SNPs. The X-axis represents different individuals, while the Y-axis represents the distribution frequency of the locus.

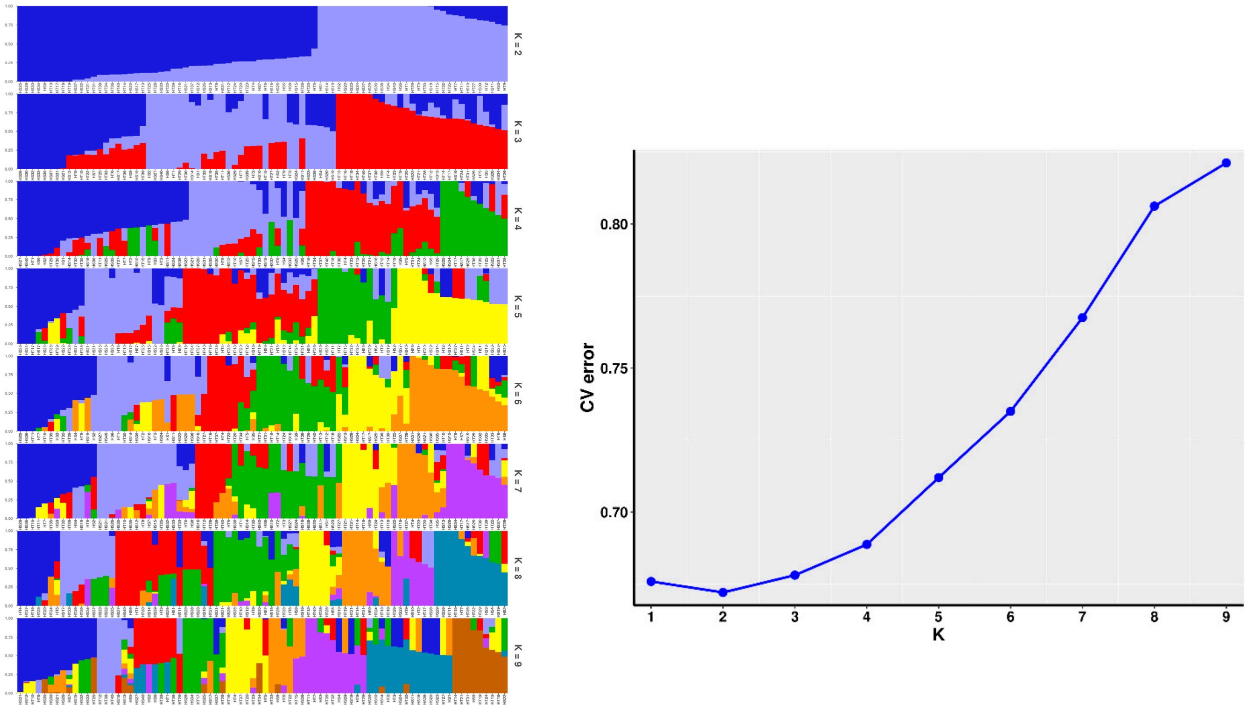

Figure S2. Cross-validation error rate line plot (left) and sample genetic composition bar chart (right).

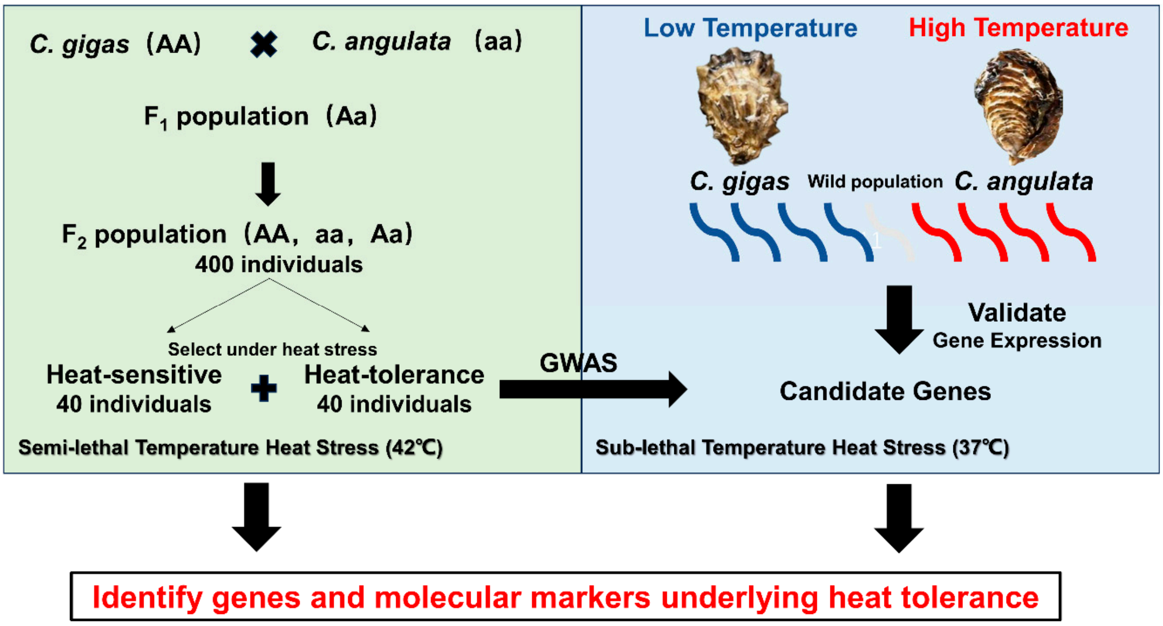

Figure S3. The flowchart of experimental design.
